# A Marine Bacterium, *Bacillus* sp. Isolated from the Sediment Samples of Algoa Bay in South Africa Produces a Polysaccharide-Bioflocculant

**DOI:** 10.3390/ijerph14101149

**Published:** 2017-09-29

**Authors:** Ncedo Ntozonke, Kunle Okaiyeto, Arinze S. Okoli, Ademola O. Olaniran, Uchechukwu U. Nwodo, Anthony I. Okoh

**Affiliations:** 1 SAMRC Microbial Water Quality Monitoring Centre, University of Fort Hare, Private Bag X1314, Alice 5700, South Africa; nntozonke@ufh.ac.za (N.N.); unwodo@ufh.ac.za (U.U.N.); aokoh@ufh.ac.za (A.I.O.); 2 Applied and Environmental Microbiology Research Group (AEMREG), Department of Biochemistry and Microbiology, University of Fort Hare, Private Bag X1314, Alice 5700, South Africa; 3 GenØK-Centre for Biosafety, Forskningsparken i Breivika, Postboks 6418, 9294 Tromsø, Norway; aso023@post.uit.no; 4 Department of Microbiology, School of Life Sciences, University of KwaZulu-Natal, Private Bag X54001, Durban 4000, South Africa; olanirana@ukzn.ac.za

**Keywords:** marine environment, *Bacillus* sp., bioflocculants, polysaccharide, flocculation

## Abstract

Bioflocculants mediate the removal of suspended particles from solution and the efficiency of flocculation is dependent on the characteristics of the flocculant. Apart from the merits of biodegradability and harmlessness, bioflocculants could be viable as industrially relevant flocculants as they are a renewable resource. Additionally, the shortcomings associated with the conventionally used flocculants such as aluminium salts and acrylamide polymers, which include dementia and cancer, highlight more the need to use bioflocculants as an alternative. Consequently, in this study a marine sediment bacterial isolate was screened for bioflocculant production. Basic local alignment search tools (BLAST) analysis of 16S ribosomal deoxyribonucleic acid (rDNA) sequence of the bacterial isolate showed 98% similarity to *Bacillus thuringiensis* MR-R1. The bacteria produced bioflocculant optimally with inoculum size (4% *v/v*) (85%), glucose (85.65%) and mixed nitrogen source (urea, ammonium chloride and yeast extract) (75.9%) and the divalent cation (Ca^2+^) (62.3%). Under optimal conditions, a maximum flocculating activity of over 85% was attained after 60 h of cultivation. The purified polysaccharide-bioflocculant flocculated optimally at alkaline pH 12 (81%), in the presence of Mn^2+^ (73%) and Ca^2+^ (72.8%). The high flocculation activity shown indicates that the bioflocculant may contend favourably as an alternative to the conventionally used flocculants in water treatment.

## 1. Introduction

Flocculation is a process whereby suspended particles are agglomerated into larger floc. Flocculation processes have applications in pharmaceutical, dredging, dairy, fermentation, and other downstream processes [1]. Flocculating agents are grouped into inorganic, organic and biologically- derived flocculants termed bioflocculants [2]. Inorganic and organic synthetic flocculants include aluminium sulphate, polyaluminum chloride, and derivatives of acrylamide and imine polymers which have dominated and, continue to dominate as flocculation mediators in the flocculation stage of water treatment processes [2,3,4].

These flocculating agents are noted as cost-effective and they mediate flocculation with high efficiency hence, their dominance as the conventionally used flocculants. Nonetheless, an associated shortfall has been their implication in Alzheimer’s disease [5], cancer and other debilitating illnesses [6,7]. Additionally, the polymers of acrylamide and imine are recalcitrant to biodegradation [8], with all degradation attempts resulting in the reduction of the polymers to monomeric units which eventually may percolate into sediments or water beds and perhaps, seep into underground waters [9]. The presence of monomeric units of acrylamide and imine polymer derivatives in the environment, constitutes a serious public health concern [10].

Conversely, bioflocculants have not been associated with any adverse effects [11,12,13]. The enormous advantages associated with bioflocculants makes them interesting; although, industrial application with respect to the water treatment process has been limited by the generally low flocculating activity of bioflocculants from several microbial species, low bioflocculant yields, and the high cost of bioflocculant production [8,14,15]. The imperative of identifying bioflocculants has propelled the exploration of extreme environments seeking microbial species with enhanced bioflocculant production potential and as well with high flocculation efficiencies.

Several terrestrial and aquatic environments have been explored and microbes from various taxonomic genera including Streptomyces, Brachybacteria and Cellulomonas [9,16]; amongst the actinobacteria species *Vagococcus*, *Arthrobacter*, *Scenedesmus* and *Bacillus* [11,17,18,19], among others, have all been found to produce bioflocculants. The compositions of some of the identified bioflocculants have included polysaccharides, proteins, uronic acids, sugar conjugates and proteins [20,21,22]. The chemical compositions and flocculating efficiency of bioflocculants depends on some factors, including the nature of the environment in which bioflocculant-producing microorganisms were isolated, the media compositions in which the microorganisms are cultivated, the functional groups and molecular weight of the bioflocculant [23].

The marine environment remains a potential source of microbes with novel metabolites, and the exploitation of this ecosystem through searching for microbes with novel metabolites is still very important [24,25]. Therefore, the continued exploration of different habitats for novel microbial species with improved bioflocculant production potential has been a focus of research in this field. In this study, a bioflocculant-producing bacterial strain was isolated from sediment samples from the marine environment in the Eastern Cape, South Africa; the culture conditions influencing bioflocculant production was optimized and the bioflocculant produced was purified and characterized afterwards.

## 2. Materials and Methods

### 2.1. Screening for Bioflocculant-Producing Bacteria

Sediment samples were collected from Algoa Bay in the Eastern Cape Province of South Africa and processed according to the description of Jensen et al. [26] with some modifications. A wet sample (0.5 g) was diluted with sterile seawater (5 mL). The suspension was agitated and allowed to settle for 60 s, then an aliquot of the upper phase (100 μL) was inoculated onto the surface of R2A agar plates, spread with a sterile glass rod and incubated for 96 h at 28 °C. The distinct isolates were selectively picked and streaked onto nutrient agar plates to purify them. The bacterial isolate was activated by inoculating glycerol stock (5 μL) into sterile broth (5 mL) composed of beef extract (3 g), tryptone (10 g) and NaCl (5 g) in sterile filtered sea water (1 L), and incubated aerobically for 24 h at 28 °C in a rotary shaker at 120 rpm. The growth medium was composed of glucose (20 g), K_2_HPO_4_ (5 g), KH_2_PO_4_ (2 g), MgSO_4_·7H_2_O (0.3 g), yeast extract (0.5 g), urea (0.5 g), and (NH_4_)_2_SO_4_ (0.3 g) in filtered marine water (1 L) [20]. A set of 500 mL flasks containing fermentation medium (200 mL) were aseptically inoculated with activated culture 2% *v/v* (4 mL), after adjusting to cell density of about 1.5 × 10^8^ CFU/mL and incubated at 28 °C, 160 rpm for 72 h. After the cultivation period, the fermented broth was centrifuged (4000 rpm, 30 min, 15 °C) to separate the bacterial cells and the cell-free supernatant was assessed for flocculating activity [3].

### 2.2. Determination of Flocculating Activity of Bioflocculant

The flocculating activity was determined using the method described by Kurane et al. [27], in which Kaolin clay (Merck, Darmstadt, Germany) was chosen as the suspended solid. Culture supernatant (2 mL) and CaCl_2_ (3 mL, 1% *w/v*) were added into kaolin clay suspension (100 mL, 4 g/L) in a 250 mL flask, gently shaken and transferred into a 100 mL measuring cylinder and later left standing for 5 min. The control was prepared following the same procedure but the bioflocculant was replaced by freshly prepared medium. The turbidity of the upper phase was measured with a spectrophotometer (Helios Epsilon, New York, NY, USA) at 550 nm and the flocculating activity was estimated as follows:

*Flocculating rate* = {(*A* − *B*)/*A*} × 100%

where *A* is the optical density of the control at 550 nm; and *B* is the optical density of the sample at 550 nm. All experiments were performed in triplicates for the mean calculation. The bioflocculant-producing bacterial isolate was preserved in 20% *v/v* glycerol stock at −80 °C as part of the culture collection of the Applied and Environmental Microbiology Research Group (AEMREG, University of Fort Hare, Alice, South Africa).

### 2.3. Identification of Bioflocculant-Producing Bacteria

The bioflocculant-producing bacterial isolate was identified by the molecular technique described by Cook and Mayers [28], and Nwodo et al. [2]. The bacterial 16S rDNA gene was amplified by polymerase chain reaction (PCR) followed by sequence analysis of the amplified gene. The bacterial DNA was extracted through the boiling method and the PCR amplification was carried out in 50 μL reaction volume containing 2 mM MgCl_2_, 2 U Supertherm Taq polymerase, 150 mM of each dNTP, 0.5 mM of each primer (F1: 59-AGAGTTTGATCITGGCTCAG-39; I = inosine and primer R5:59-ACGGITACCTTGTTACGACTT-39) and 2 mL of the template DNA. Primer F1 and R5 bind to base positions 7–26 and 1496–1476 of the 16S DNA gene of *Streptomyces ambofaciens* ATCC23877, respectively [28]. The PCR conditions included the following steps: initial denaturation (96 °C for 2 min), 30 cycles of denaturation (96 °C for 45 s), annealing (56 °C for 30 s) and extension (72 °C for 2 min), and a final extension (72 °C for 5 min). Gel electrophoresis of PCR products was carried out on 1% agarose gels to confirm that a fragment of the correct size had been amplified. Automated sequencing of the 16S rDNA gene of the bacterial isolate was performed using the SCE2410 genetic analysis system (Spectrumedix, Fullerton, CA, USA,) equipped with 24 capillaries. The sequencing reactions were performed according to the manufacturer’s instructions, using the Big Dye version 3.1 dye terminator cycle sequencing kit (Applied Biosystems, Foster city, CA, USA) and 27 F primer (5’-AGAGTTTGA TCMTGGCTCAG-3’). The sequences were edited manually based on the most similar sequences, and the results obtained were aligned with published 16S rDNA sequences in the GenBank through a BLAST sequence tool from the National Centre for Biotechnology Information (NCBI) Database (https://www.ncbi.nlm.nih.gov).

### 2.4. Effect of Inoculum Size on Bioflocculant Production

Inoculum sizes of 1% (0.5 mL), 2% (1.0 mL), 3% (1.5 mL), 4% (2.0 mL) and 5% (2.5 mL) (*v/v*) in 50 mL of fermentation medium were assessed for bioflocculant production. These cultures were incubated at 160 rpm, 28 °C for 72 h. After the incubation period, 2 mL of the fermentation broth was centrifuged (4000 rpm, 30 min, 15 °C) and the supernatant was assessed for flocculation activity [2].

### 2.5. Effect of Carbon, Nitrogen and Cation Sources on Bioflocculant Production

To examine the effect of carbon, nitrogen and cation sources on bioflocculant production, carbon, nitrogen and cation sources were varied to test their effect. The carbon sources (20 g/L) investigated were; glucose, sucrose, fructose, starch, lactose and maltose while the nitrogen sources (1.3 g/L) assessed were; ammonium chloride, urea, tryptone, peptone and yeast extract and Al^3+^, Fe^3+^, K^+^, Na^+^, Li^+^, Mg^2+^ and Mn^2+^ at the concentration (0.3 g/L) were used individually to replace Mg^2+^ in the basal salt medium used for screening the bacteria for bioflocculant production. The assessments were conducted in accordance to the method described by Lachhwani [29].

### 2.6. Effect of Initial Medium pH on Bioflocculant Production

To assess the effect of initial medium pH on bioflocculant production, 50 mL fermentation medium contained in 250 mL capacity flasks were adjusted with 0.1 M NaOH and 0.1 M HCl to pH values 2–12. Each flask was aseptically inoculated with activated culture (2 mL) 4% (*v/v*) of fermentation medium. These cultures were incubated at 160 rpm, 28 °C for 72 h.

### 2.7. Time Course of Bioflocculant Production

For the time course experiments, an optimum medium composition was used, which included glucose (20 g), K_2_HPO_4_ (5 g), KH_2_PO_4_ (2 g), NaCl (0.3 g), yeast extract (0.5 g), urea (0.5 g), and (NH_4_)_2_SO_4_ (0.3 g) in filtered marine water (1L) [3].

Saline solution (50 mL, 0.85%) was used to prepare the suspension of the bacterial isolate. The optical density was measured by taking the suspension (100 μL) in distilled water (1 mL) and gradually readjusting the OD_660_ to 0.1 [20]. The standardized bacterial suspension (4 mL) was inoculated into production medium (200 mL) contained in 500 mL flasks and incubated in a rotary shaker at 160 rpm, 28 °C for over 80 h. At 24 h intervals, an aliquot of the fermented broth (10 mL) was withdrawn and 2 mL of cell-free supernatant were used to determine the flocculating activity in accordance with the method of Kurane et al. [27]. The bacterial growth was monitored by measuring the optical density (OD) at OD_660_ and the bacterial counts were determined by a standard spread plate technique. The pH of the culture broth was also monitored simultaneously with a pH meter (Basic20 pH meter, Crison Instruments, Barcelona, Spain).

### 2.8. Extraction and Purification of Bioflocculant

Extraction and purification of the bioflocculant were carried out following the methods described elsewhere using media formulation based on the pre-determined optimum culture conditions [12]. After 60 h of fermentation, the fermented broth was centrifuged at 4000 rpm for 30 min. In order to remove insoluble substances [13,17], one volume of sterile distilled water was added to the supernatant and centrifuged at 4000 rpm for 30 min. Subsequently, two volumes of ethanol were added to the supernatant and the mixture allowed standing at 4 °C overnight. The precipitate was collected by centrifugation (4000× *g* for 15 min), and vacuum dried. The crude extract was weighed and dissolved in 100 mL of distilled water and one volume of a mixed solution of chloroform and *n*-butyl alcohol (5:2 *v/v*) was added. The mixture was agitated for 60 s and left standing for 12 h at room temperature. The precipitate was re-suspended in 100 mL distilled water and later dialysed against distilled water overnight. Two volumes of ethanol were added to the dialyzed solution (100 mL) and the precipitate recovered was dissolved in 50 mL of distilled water and vacuum dried.

### 2.9. Effect of Temperature, Cations and pH on the Flocculating Activity

The flocculating activity of the purified bioflocculant was assessed using kaolin clay suspension as the test material to simulate the turbidity of surface water [27]. To evaluate the effect of temperature on flocculating activity of the bioflocculant, the bioflocculant solution (2 mL) was heated at different temperatures (50, 60, 70, 80 and 90 °C) for 1 h. The effect of pH on the flocculating activity was determined by varying the pH of the kaolin clay suspension from 3 to 12. Similarly, the cation sources were varied as well from monovalent cations (K^+^, Na^+^ and Li^+^), divalent cations (Mg^2+^, Mg^2+^ and Mn^2+^), and trivalent cations (Al^3+^ and Fe^3+^), all other parameters were kept constant [30].

### 2.10. Characterization of Purified Bioflocculant

The protein content of the purified bioflocculant was determined using the Bradford method [31]. Total sugar content was measured using the phenol-sulphuric acid protocol as described by Chaplin and Kennedy [32].

### 2.11. Statistical Analysis

All data were treated in replicates and the standard deviation of the mean values was taken. Data were subjected to one-way analysis of variance (ANOVA) using MINITAB Student Release 12 statistical package for Windows 95/98 NT (Minitab Inc., State College, PA, USA, 2007).

## 3. Results and Discussion

### 3.1. Screening and Identification of Bioflocculant-Producing Bacteria

The bacteria used in this study were isolated from sediment samples of Algoa Bay in the Eastern Cape of Province, South Africa that were screened for bioflocculant production by investigating their potential to flocculate a kaolin clay suspension. The bacteria showed good bioflocculant production potential with a flocculating activity of over 60% for kaolin clay suspension. Furthermore, the nucleotide sequence of its 16S rDNA gene showed 98% similarity to *Bacillus thuringiensis* MR-R1. Subsequently, the production of bioflocculant by the test bacteria was optimized through medium compositions and fermentation conditions.

### 3.2. Effect of Inoculums Size on Bioflocculant Production

**Figure 1:**
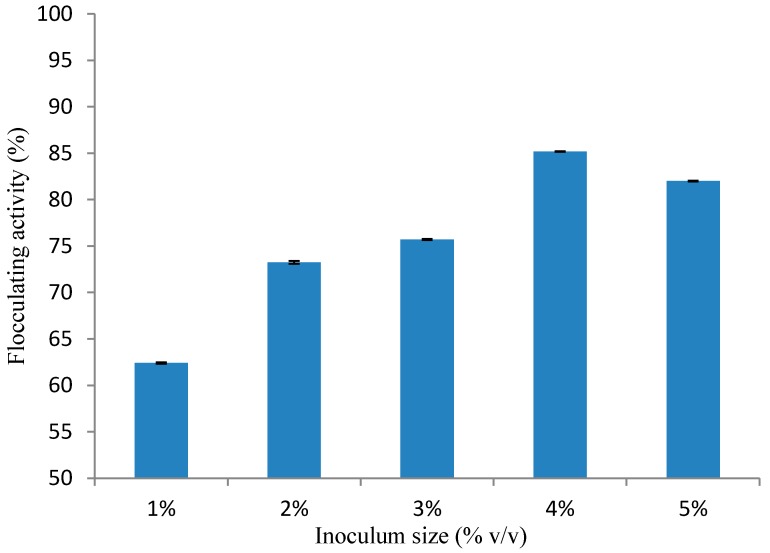
Effect of Inoculum Size on Bioflocculant Production by *Bacillus* sp.

The effects of different inoculum sizes on bioflocculant production by *Bacillus* sp. was investigated. Inoculum sizes of 1%, 2%, 3%, 4% and 5% (*v/v*) production medium were assessed, representing 0.5, 1.0, 1.5, 2.0 and 2.5 mL in 50 mL, respectively. From Figure 1, it can be observed that the bioflocculant production by the tested strain (*Bacillus* sp.) was inoculum size-dependent, with the highest flocculating activity (85.1%) attained at 4% (*v/v*).

After the optimum inoculum size was reached, any further increase in inoculum size resulted into a decrease in the flocculating activity, hence, inoculum size of 4% (*v/v*) was used in the subsequent experiments. Previous studies have proven that inoculum size plays an essential role in bioflocculant production, as a small inoculum size prolongs the lag phase during growth, whereas too large an inoculum size make the niche of strains overlap and restrains bioflocculant production due to the limit of nutrient allocation [33].

On the other hand, our present findings did not match previous studies. For example, a bioflocculant produced by Virgibacillus species was produced optimally at an inoculum size of 2% (*v/v*) [20], while, an inoculum size of 5% (*v/v*) was observed to be optimal for bioflocculant production by Klebsiella mobilis strain [34]. Xiong et al. [15] and Makapela et al. [22] observed the same phenomenon where Bacillus licherniformis and Bacillus pumilus optimally produced a bioflocculant at an inoculum size of 4% (*v/v*).

### 3.3. Effect of Carbon, Nitrogen and Cation Sources on Bioflocculant Production

The ability of bacteria to produce secondary metabolites could be influenced by the media composition and fermentation conditions [4]. Since the carbon source plays a very significant role in growth, particularly in extracellular polysaccharide production. Hence, in this present study, various carbon sources were assessed in order to improve the bioflocculant production. Table 1 shows the effect of various carbon sources on bioflocculant production. Glucose was observed to be the best carbon source for bioflocculant production by the tested strain (*Bacillus* sp.) with a flocculating activity of 85.65%, followed by sucrose (60.6%). On the other hand, lactose, maltose and starch poorly supported bioflocculant production with flocculating activities of 22.45%, 18.5% and 10.2%, respectively. Hence, glucose was used in the subsequent experiments. From our findings, we observed that though a fructose-containing medium supported bacteria growth it was unable to activate the gene implicated in bioflocculant production and hence, no flocculating activity was observed compared with glucose-containing medium.

Glucose has been well documented in previous studies as the best organic sole carbon source [20]. For example, *Streptomyces* sp. Gansen produced bioflocculant optimally when glucose was the sole carbon source [2]. Furthermore, numerous bioflocculant-producing bacteria such as *Serratia ficaria* species and a mixed culture of *Methylobacterium* sp. Obi and *Actinobacterium* sp. Mayor prefer organic carbon sources for bioflocculant production [35,36]. On the contrary, Zhang et al. [3] revealed that glucose inhibited cell growth in *Sorangium cellulosum* during bioflocculant production.

Nitrogen sources play a crucial role in the production of bioflocculants [4]. The effect of organic, inorganic or combined nitrogen sources (urea, (NH_4_)_2_SO_4_ and yeast extract) on bioflocculant production by the tested strain (*Bacillus* sp.) was examined. Among the sole nitrogen sources, peptone had the highest flocculating activity of 63.6%. However, the combined nitrogen sources gave the highest activity of 75.9%. Low flocculation rates were observed on media supplemented with yeast extract (33%), urea (27.7%) and (NH_4_)_2_SO_4_ (35.25%). In agreement with our findings, Cosa et al. [20] observed that complex nitrogen substrate consisting of urea, yeast extract and (NH_4_)_2_SO_4_ enhanced bioflocculant production than the other sole nitrogen sources tested. Okaiyeto et al. [4] reported that higher flocculating activity (86.35%) was obtained when mixed nitrogen sources was used for bioflocculant production by *Bacillus* sp. AEMREG7, whereas, Gong et al. [35] observed 97% flocculating activity from the bioflocculant produced by *Serratia ficaria* in beef extract and urea supplemented medium as nitrogen source. In this present study, on optimizing the media compositions, we observed that alteration in the carbon (glucose) and nitrogen sources [urea + yeast extract + (NH_4_)_2_SO_4_] in the initial screening medium with complex nitrogen source (urea, yeast extract and (NH_4_)_2_SO_4_) and glucose as sole carbon source, was used in subsequent experiments because alteration in the media composition decreased the bioflocculant production by the test bacteria.

The effect of different cations on bioflocculant production was investigated and out of eight cation sources tested, Ca^2+^, Mn^2+^ and Al^3+^ enhanced bioflocculant production with the following activities: 62.3%, 65.1% and 69.8%, respectively.

### 3.4. Time Course of Bioflocculant Production

**Figure 2:**
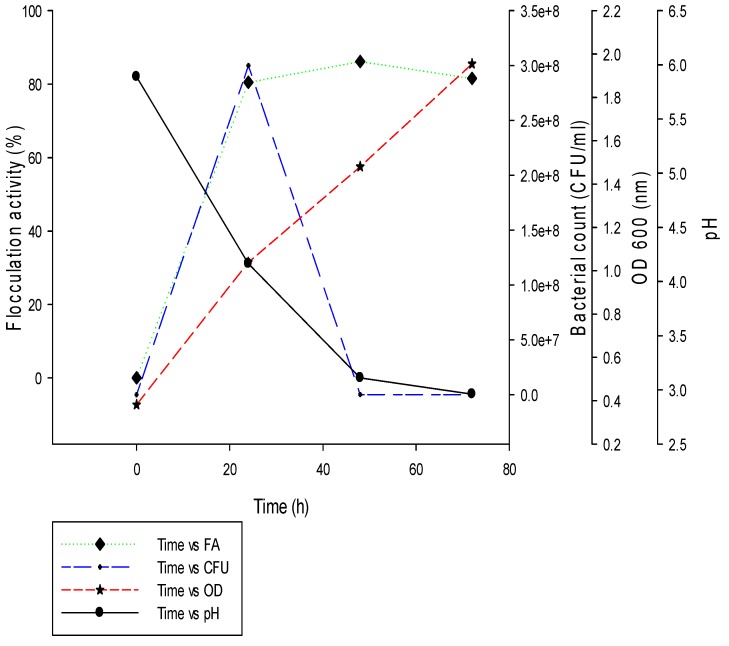
Time Course Profile of Bioflocculant Production by *Bacillus* sp.

Several factors influence bioflocculant production and thereby influence the flocculation process. These factors include: culture time, cell growth and pH. Amongst these factors culture time may influence the production, distribution and flocculating capabilities of the bioflocculant [12]. Although, culture time for flocculant release into the medium and its activity may differ with different microorganisms, most are produced biosynthetically. Optimum culture conditions were used for the time course experimentation over 80 h of cultivation period (Figure 2).

There was a rapid exponential increase in bioflocculant production starting from the first 24 h and subsequently reached a peak after 60 h (85%) at pH 3. Thereafter, a decline or levelling of cell growth (bacterial counts) was observed and thus, this decline may be due to cell autolysis and enzymatic activity [14]. From the cell growth curve pattern, it was observed that the cell growth was parallel to the flocculating activity from 0 to 24 h. This indicates that the bioflocculant was produced during the growth of the bacteria not by cell autolysis [22]. A gradual decrease in pH was observed from 5.89 after 24 h to 2.96 after 72 h. The decrease in pH with increase in cultivation time may be due to the production of organic acids from the metabolism of glucose or due to the production of organic acid as part of the components of the produced bioflocculant [17]. A slow decrease in flocculating activity was observed after 60 h of incubation, this observed decrease in activity may be attributed to the action of a bioflocculant-degrading enzyme being produced by the microorganism. Similarly, Piyo et al. [37] observed the same phenomenon whereby *Bacillus* sp. Gilbert produced a bioflocculant with flocculating activity increasing with the increase of cultivation time. The same phenomenon was observed by Cosa et al. [12] where *Virgibacillus* sp. Rob showed highest flocculating activity at day 4. Furthermore, *Cobetia* sp. produced a bioflocculant during the exponential phase [38]. After 60 h of incubation, flocculating activity began to decrease, while cell growth decreased gradually after 50 h. The decrease in flocculating activity is due to cell autolysis and/or presence of a bioflocculant-degrading enzyme [20,38].

### 3.5. Factors Affecting Flocculating Activity of the Purified Bioflocculant

#### 3.5.1. Effect of Bioflocculant Dosage

**Figure 3:**
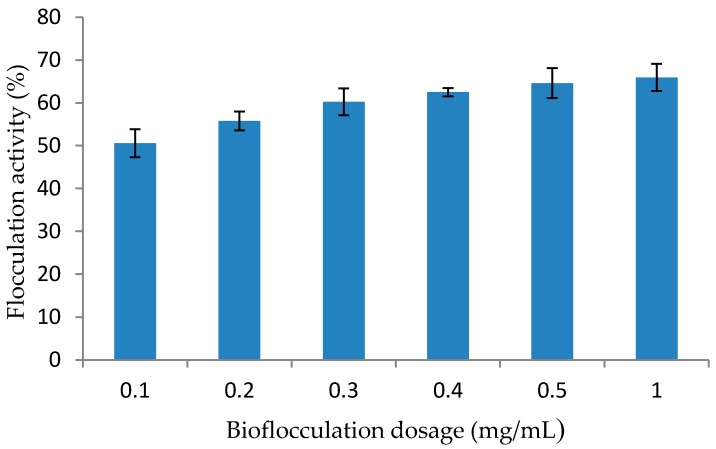
Effect of Bioflocculant Dosage on Flocculating Activity of the Purified Bioflocculant.

The appropriate bioflocculant dosage to be used for subsequent experiments was determined by investigating different bioflocculant dose ranging from 0.1 to 0.5 mg/mL (Figure 3). The aim of the bioflocculant dosage experiment was to determine the lowest bioflocculant concentration to be used while attaining the highest flocculating efficiency [19,22]. Bioflocculant dosage is strain dependant and hence, bioflocculant produced in this present study attained an optimal activity at 0.5 mg/mL.

Contrary to our present findings, Okaiyeto et al. [13] reported optimal flocculating activity at 0.1 mg/mL by a bioflocculant produced by a consortium of *Halomonas* sp. Okoh and *Micrococcus* sp. Leo. Ntsaluba et al. [36] documented a dosage of 2 mg/mL for bioflocculant produced by a consortium of *Methylobacterium* sp. Obi and *Actinobacterium* sp. Mayor, while Wang et al. [39] found that a mixed culture of *Rhizobium radiobacter* F2 and *Bacillus sphaeicus* F6 produced a bioflocculant which had a maximum activity (96.21%) at 12 mg/mL. These results revealed that the bioflocculant produced by *Bacillus* sp. showed high flocculating activity at very low dosage, which should be beneficial to industrial scale applications as low dosage reduce treatment costs [22]. In case of bioflocculant (MBF-W7) produced by *Bacillus* sp., the highest flocculating activity was observed at 0.2 mg/mL; subsequently, the flocculation rate decreased below or above this dosage value [40].

#### 3.5.2. Effect of Cation and Temperature on the Flocculating Activity of the Purified Bioflocculant

As a strategy to produce biofloculants in a large industrial scale, it is highly imperative to isolate bacteria with high bioflocculant-producing capability as well as with high flocculating efficiency [4]. Cations play a crucial role in the flocculation efficacy of a bioflocculant. Cations have the ability to neutralize negatively charged functional groups of both bioflocculant molecules and suspended particles, thereby increasing the adsorption of the bioflocculant onto the suspended particles [4].

In this present study, out of eight cations investigated, only Mn^2+^ and Fe^3+^ inhibited flocculation among others. From Table 2, Al^3+^, Ca^2+^ and Mg^2+^ had the highest flocculating with 73.9%, 72.8% and 70.9%, respectively. Aluminium salts have been frequently used in drinking water and wastewater treatment due to their high flocculating efficiency and cost-effectiveness. Nevertheless, residual aluminium concentrations in treated water impose health problems [21]; consequently, in this present study, Ca^2+^ was used as a flocculation aid. Furthermore, Nwodo and Okoh [16] reported similar findings by *Cellulomonas* sp. Okoh. The flocculating activity of bioflocculant produced by *Bacillus* sp. AEMREG7 was greatly enhanced in the presence of Ca^2+^ as well [21]. Valence plays a crucial role in the flocculation efficiency, as observed by Okaiyeto et al. [13], the flocculating activity of the bioflocculant produced by *Halomonas* sp. and *Micrococcus* sp. optimally flocculated in the presence of Al^3+^ and Ca^2+^.

In most studies reported in the literature, flocculation processes have been enhanced with cations of high valence, as they stimulate more flocculation by neutralizing and forming bridges that bind kaolin [41]. In this present study, it was observed that flocculating activity was more enhanced by trivalent and divalent cations except for Mn^2+^ and Fe^3+^ (Table 2). Li et al. [24] emphasized that cations have the ability to neutralize negatively charged functional groups of both bioflocculant molecules and suspended particles, thereby increasing the adsorption of the bioflocculant onto the suspended particles, hence, monovalent cations weakly neutralize these negative charges in comparison to cations of higher valences. Fe^3+^ might possibly alter the surface charge of kaolin surfaces and cover the adsorb sites. The competition of the positively charged particles and less adsorb sites might induce the antagonist effect of Fe^3+^ that resulted into poor flocculation [42]. This implies that the electrostatic repulsive forces between Fe^3+^ and charged groups of the bioflocculant will be higher as compared with divalent cations and Al^3+^ and this consequently leads to the inhibition of flocculating activity of the bioflocculant in the presence of Fe^3+^. Excessive adsorption of the ions might be the cause of inhibition of the flocculation process in the presence of Fe^3+^ [42].

The thermal stability of bioflocculant is an important property for its commercial exploitation. Several studies have documented different organisms that produced thermal stable bioflocculants [13,43,44,45,46]. The thermal stability of the purified bioflocculant was examined at 50, 60, 70, 80, and 90 °C for 1 h at each temperature. Table 2 shows the effect of temperature on flocculating activity of the bioflocculant produced by *Bacillus* sp. The bioflocculant showed permanence to heat as an increase in flocculating activity was observed up to 90 °C. Okaiyeto et al. [40] reported that the exhibition of thermal stability by bioflocculants may be characteristic of their polysaccharide backbone.

#### 3.5.3. Effect of pH on Flocculating Activity of Purified Bioflocculant

**Figure 4:**
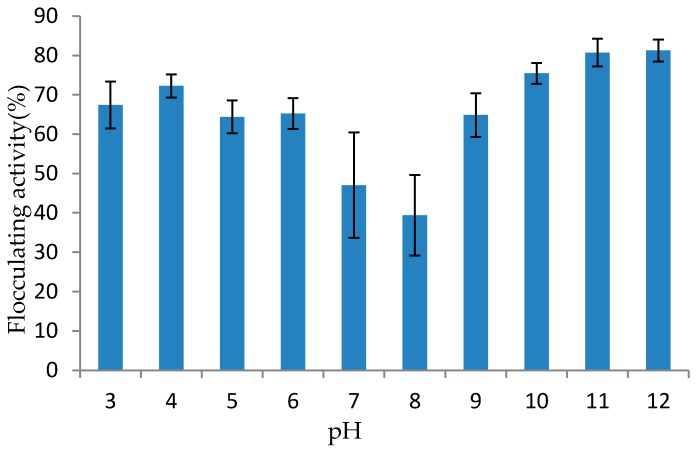
Effect of pH on Flocculating Activity of Bioflocculant Produced by the Tested Strain (*Bacillus* sp.).

Flocculating activity of purified bioflocculant was greatly influenced by change in pH of the reaction mixture. It was observed that the highest flocculation rate was at the extremes, with maximum activity at pH 12 (81.25%) (Figure 4). This implies that the bioflocculant can be used to treat or used at various pH ranges especially at acidic and alkaline conditions. The surface area of the purified bioflocculant increased in the extreme pH regimes and this aided its flocculation efficiency. In this current study, the bioflocculant produced by *Bacillus* sp. flocculated well over a wide range of pH values, the lowest being only 39% and 47%. The decrease in flocculating activity between pH 6 and 7, may be due to the hydroxyl ions (OH^−^) adsorbed at neutral medium (pH 6 and 7) may interfere with the complex formed between bioflocculant produced and the kaolin particles mediated by Ca^2+^, thereby resulting in destabilization of the suspended particles [40]. From our findings, pH stability of the purified bioflocculant portends good industrial applicability. Alteration of pH may perhaps ultimately alter the bioflocculant charge status and surface characteristics of suspended materials which consequently changes the flocculating ability [46]. The findings of Zaki et al. [47] collaborate with our results in which the purified bioflocculant MBF-5 produced by *Klebsiella pneumonia* maintained higher flocculating activity (90–95%) under acidic condition (pH 2–5) and alkaline condition (pH 8–11). Contrary to our findings, Aljuboori et al. [48] observed that the bioflocculant IH-7 produced by *Aspergillus flavus* showed over 90% flocculating rate at a wide pH range of 3–7. The bioflocculant was only suitable at acidic and neutral conditions.

#### 3.5.4. Fourier Transform Infrared (FTIR) Spectroscopy of the Purified Bioflocculant

**Figure 5:**
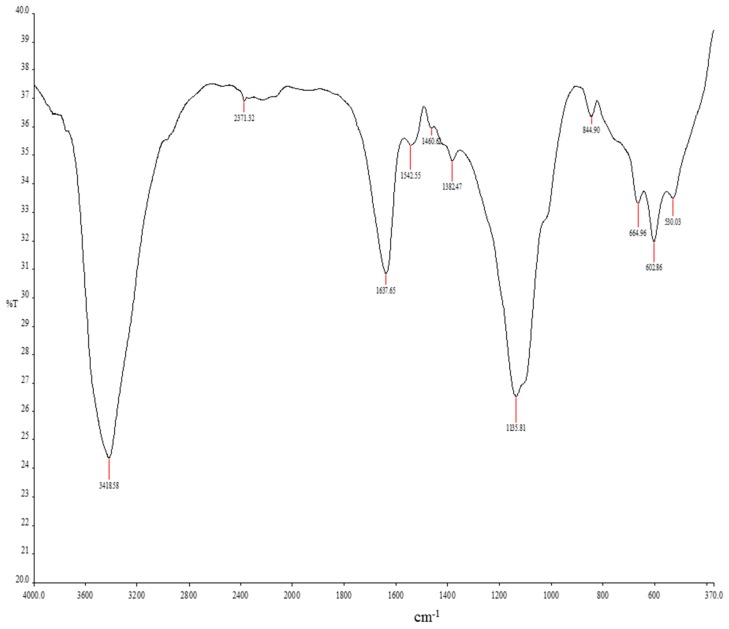
FTIR Spectroscopy of Purified Bioflocculant Produced by the Tested Strain (*Bacillus* sp.).

A broad stretching peak was observed at 3418.54 cm^−1^ which indicated the presence of hydroxyl and amine groups (Figure 5).

This may also be as a result of vibration of OH^−^ or NH_2_ groups present in the sugar ring [13,15], and the weak peaks at 844.90 cm^−1^ reveal the presence of sugar derivatives whereas, the one at 1460.62 cm^−1^ indicated the presence of uronate in the polysaccharide. The vibration peak at 1135.81 cm^−1^ corresponds to the C-O stretching in alcohols and this further suggests the presence of OH group in the bioflocculant molecule [2,43]. Peaks at 1637.65 and 1460.62 cm^−1^ from the polymeric and dimeric OH stretches of phenol or tertiary alcohol bends indicate the presence of carboxyl and hydroxyl groups. The strong absorption peak observed at 530.03 cm^−1^ is known to be a typical characteristic of sugar derivatives. The infrared spectrum of the bioflocculant thus indicated the presence of carboxyl, hydroxyl and amino groups which might be responsible for the high flocculating efficiency observed in this study [49].

## 4. Conclusions

The adverse effects of chemical flocculants such as potential carcinogenicity, non-biodegradability, neurotoxicity and neurodegenerative effects, led us to explore the marine milieu seeking innocuous and biodegradable microbial flocculants. In this present study, a *Bacillus* sp. was isolated from the sediments of Algoa Bay in South Africa and its potential for bioflocculant production was investigated. The bacterium showed a flocculating activity over 60% on initial screening. The bioflocculant optimally flocculated when glucose, mixed nitrogen sources (yeast extract, urea, and (NH_4_)_2_SO_4_ and Ca^2+^ were used as carbon, nitrogen and cation sources, respectively. The bioflocculant had strong flocculating activity over a wide range of pH, both at acidic and alkaline pH values, with low dosage requirements. The flocculating activity of the bioflocculant was stimulated in the presence of Ca^2+^. The presence of hydroxyl, amino and carboxyl groups as the main functional groups in the molecular chain of the bioflocculant might be responsible for its high flocculating efficiency. This bioflocculant could serve an alternative to replace the harmful and non-degradable chemical flocculants that are widely used in wastewater treatment, hence, making it candidate for further research and possible development for industrial-scale application.

## Figures and Tables

**Table 1:** Effect of Carbon, Nitrogen and Cation Sources on Bioflocculant Production by *Bacillus* sp.

Carbon Source	Fructose	Sucrose	Maltose	Glucose	Lactose	Starch		
FA (%)	-	60.6 ± 0.7	18.5 ± 0.9	85.65 ± 1.0	22.45 ± 2.0	10.2± 1.5		
Nitrogen Source	Peptone	Urea	Yeast extract	Tryptone	(NH_4_)_2_SO_4_	Combined		
FA (%)	63.6 ± 2.8	27.7 ± 2.9	33 ± 3.2	22.1 ± 1.8	35.25 ± 0.5	75.9 ± 1		
Cations	Fe^3+^	Al^3+^	Ca^2+^	Mn^2+^	Mg^2+^	Li^+^	Na^+^	K^+^
FA (%)	5 ± 0.9	69.8 ± 2.8	62.3 ± 3.1	65.1 ± -	61.1 ± 1.1	23.9 ± 2	13 ± 2.8	13 ± 2.8

FA represents flocculating activity and combined nitrogen source (Urea + (NH_4_)_2_SO_4_ + yeast extract).

**Table 2:** Effect of Temperature and Cation on the Flocculating Activity of Purified Bioflocculant.

**Temp (°C)**	**50**	**60**	**70**	**80**	**90**			
FA (%)	66.6 ± 0.64	70 ± 1.2	70.8 ± 0.0	72.2 ± 1.5	69.4 ± 2.3			
**Cations**	**Al^3+^**	**Fe^3+^**	**Ca^2+^**	**Mn^2+^**	**Mg^2+^**	**K^+^**	**Li^+^**	**Na^+^**
FA (%)	73.9 ± 3.4	8.7 ± 6.8	72.8 ± 4.3	-	70.9 ± 2.1	65 ± 0.8	57.3 ± 2.6	57.2 ± 3.9

FA = Flocculating activity.
